# Validation of the Committed Action Questionnaire-8 and Its Mediating Role Between Experiential Avoidance and Life Satisfaction Among Chinese University Students

**DOI:** 10.3389/fpsyg.2021.655518

**Published:** 2021-11-24

**Authors:** Ya Li, Fei-long Yang, Chen Pan, Qian-qian Chu, Qiu-ping Tang

**Affiliations:** ^1^Department of Clinical Psychology, The Third Xiangya Hospital, Central South University, Changsha, China; ^2^School of Nursing, Hunan University of Chinese Medicine, Changsha, China; ^3^Psychosomatic Health Institute, The Third Xiangya Hospital, Central South University, Changsha, China

**Keywords:** committed action questionnaire, life satisfaction, experiential avoidance, psychometric properties, psychological flexibility

## Abstract

**Background:** Committed action is one of the core processes of psychological flexibility derived from acceptance and commitment therapy. It has not been widely investigated in mainland China as appropriate measures are lacking. The current study aimed to validate a Chinese (Mandarin) version of the Committed Action Questionnaire (CAQ-8) in a non-clinical college sample and to explore whether committed action would have a mediating effect in the association between experiential avoidance (EA) and life satisfaction.

**Methods:** We translated the CAQ-8 into Chinese (Mandarin). A total of 913 Chinese undergraduates completed a set of questionnaires measuring committed action, EA, mindful awareness, anxiety, depression, stress, and life satisfaction. For test–retest reliability, 167 respondents completed the CAQ-8 again 4 weeks later.

**Results:** The entire scale of CAQ-8 (Mandarin) and two subscales showed adequate internal consistency and acceptable test–retest reliability. Confirmatory factor analyses confirmed the two-factor structure and the convergent and criterion validity were acceptable. Committed action was correlated with less EA, more mindful awareness, less depressive symptoms, less anxiety, less stress, and more life satisfaction. In bootstrap mediation analyses, committed action partially mediated the association between EA and life satisfaction.

**Conclusion:** The results suggest that the CAQ-8 (Mandarin) is a brief, psychometrically sound instrument to investigate committed action in Chinese populations, and the relationship between EA and life satisfaction was partially explained by committed action. This study provides new information about the usefulness of CAQ-8 and supports the assumption that committed action may be considered a promising factors for improving life satisfaction who have involved in EA among an educated non-clinical population.

## Highlights

- The Committed Action Questionnaire (CAQ-8), a brief, psychometrically sound instrument, has now been translated successfully into Mandarin Chinese.
- The relationship between experiential avoidance and life satisfaction was partially explained by committed action.
- Greater committed action may be associated with lower levels of experiential avoidance and a higher level of life satisfaction.

## Introduction

As a third-wave cognitive behavioral therapy, acceptance and commitment therapy (ACT) has attracted much interest during the last two decades. For instance, a growing body of research has found that ACT, aiming at fostering psychological flexibility, is an effective treatment for psychological distress of university students (Muto et al., 2011; Räsänen et al., 2016; Juncos et al., 2017; Levin et al., 2017, 2020; Grégoire et al., 2018; Viskovich and Pakenham, 2020). Psychological flexibility refers to the ability to completely get into contact with the present moment and either stick to or modify behaviors according to values and situational prospects (Hayes et al., 1999, 2006). It includes six different but interconnected therapeutic subprocesses: acceptance, cognitive defusion, self-as-context, contact with the present moment, values, and committed action (Hayes et al., 1999, 2006, 2012). Many studies have shown that psychological flexibility may be a basic aspect of mental health and wellbeing, while psychological inflexibility is a risk factor for psychopathologies, such as chronic pain (Lin et al., 2018; Gentili et al., 2019), psychological distress, somatization, anxiety, or depression (Kashdan and Rottenberg, 2010; Masuda and Tully, 2012). Effective measurements to assess all six constructs are needed to obtain a better understanding of psychological flexibility and further insight into psychotherapeutic processes (Gagnon et al., 2017; Terhorst et al., 2020).

Several instruments have been developed to measure aspects of psychological flexibility, including cognitive defusion (Gillanders et al., 2014), acceptance (Bond et al., 2011), present-focused awareness (Brown and Ryan, 2003), self as context (Yu et al., 2016, 2017; Zettle et al., 2018), and values (McCracken and Yang, 2006). Committed action is also a key component (Galán et al., 2019) but has been less studied (McCracken, 2013).

Committed action, focusing on effective and flexible actions following valued life directions (McCracken et al., 2015), is characterized by balanced patterns of behavior that can flexibly incorporate failure and discomfort as part of the process of attaining a goal (McCracken, 2013). Previous studies suggest that engaging in committed action is correlated with higher levels of pain acceptance, health and functioning (McCracken, 2013), quality of life (Coutinho et al., 2019), and mental health (Åkerblom et al., 2016). It is also correlated with lower levels of psychosocial, physical, and independence-related disability (Bailey et al., 2016), pain intensity (McCracken, 2013), depression, anxiety, stress (Gagnon et al., 2017; Trindade et al., 2018), and procrastination (Gagnon et al., 2016).

There are two scales to measure committed action; the Engaged Living Scale (ELS) (Trompetter et al., 2013) and the Committed Action Questionnaire (CAQ) (McCracken, 2013), CAQ being more widely used. The original CAQ consists of 18 items, nine each of positively and negatively keyed items. A shortened version of the CAQ has been developed with eight items, which shows comparably satisfactory reliability and validity (McCracken et al., 2015). The CAQ-8 is psychometrically sound in various languages including Swedish (Åkerblom et al., 2016), French (Gagnon et al., 2017), German (Terhorst et al., 2020), Portuguese (Trindade et al., 2018), and Chinese (Cantonese) (Wong et al., 2016). However, there is no Chinese (Mandarin) version of the CAQ-8, although it is the most common and official language of China. Mandarin has a different grammatical structure, vocabulary, character pattern, and writing system compared with Cantonese (Tardif et al., 2009; Yang and Wang, 2018). Thus, validation of a Chinese (Mandarin) version of the CAQ-8 is needed. One aim of the current study was to preliminarily validate the CAQ-8 (Mandarin) in a mainland non-clinical sample.

Experiential avoidance (EA), a pathological aspect of ACT, is a maladaptive psychological tendency that occurs when individuals are relatively unlikely to accept unwanted inner experiences (thoughts, memories, feelings, or emotions) (Hayes et al., 2006). People scoring more highly on EA tend to try to escape or control their private upsetting experiences, which restricts their meaningful behaviors and activities, including their committed actions (Hayes et al., 2012). Greater EA is related to reduced health benefits and life satisfaction (Kashdan et al., 2006; Chawla and Ostafin, 2007), suggesting that EA may be an important risk factor for life satisfaction (Graham et al., 2016; Valdivia-Salas et al., 2017; Lucas and Moore, 2020). For example, if someone feels anxious in social situations, then to avoid feeling anxious they may restrict social activities, decreasing life satisfaction. In a recent study of breast cancer patients and healthy individuals, the association between EA and depressive symptomatology was partially explained by committed action (Trindade et al., 2018). Another study of college students found that EA and committed action were significant mediators of the association between anxiety and psychological quality of life (Coutinho et al., 2019). Based on these findings, it was hypothesized that EA, committed action, and life satisfaction are interrelated. A second aim of the study was to examine whether committed action mediates the correlation between EA and life satisfaction. Additionally, we hypothesized that higher EA scores would be associated with lower committed action which, in turn, would be associated with lower life satisfaction.

## Materials and Methods

### Participants

With the permission and assistance of teachers, 1,050 undergraduate students from the Hunan University of Chinese Medicine were asked to participate, providing an initial convenience sample of 930 undergraduate students who agreed to participate and gave written informed consent after being told the purpose and content of the study. All participants completed a paper version of the questionnaire which takes 30–40 min, without any reward or course credit. Ultimately, 913 participants (268 men and 645 women), aged 16–25 years (*M*_*age*_ = 18.84, SD = 1.25) provided fully completed questionnaires with no missing data, which gave a response rate of 98.2%. For test–retest validity, 180 participants were conveniently selected a second time 4 weeks later in class, when they completed the CAQ-8 (Mandarin) again. Of them, 13 participants had not been tested the first time or did not answer all the questions, so they were deleted from the analysis. The valid sample used in the test–retest analysis comprised 167 respondents. This study was approved by the Institutional Review Board of the Third Xiangya Hospital of Central South University (2020-S385).

### Instruments

#### The Mandarin Chinese Version of the Committed Action Questionnaire-8

The primary CAQ-8 includes eight items with two subscales (Kashdan and Rottenberg, 2010), one positive subscale named values persistence (VP) and one negative subscale named non-reactive behavior (NB), with four items each. Participants are asked to rate each item on a seven-point Likert scale ranging from 0 (never true) to 6 (always true). Primary research found that the total scale, VP subscale, and NB subscale of CAQ-8 have good (respectively, α = 0.87, 0.87, and 0.80) reliability (McCracken et al., 2015).

We followed the recommended guidelines (Brislin, 1970) to translate the CAQ-8 from the original English version to Mandarin Chinese. First, after gaining permission for translation from the original author, Lance M. McCracken, forward translation (English to Mandarin Chinese) was conducted by a panel of bilingual psychological researchers. Next, two psychology professors, familiar with ACT, checked and revised the translation to make it suitable for the mainland Chinese population. Then, an independent professional bilingual translator performed a back-translation. After that, the original author of the CAQ-8 then examined and reviewed the back-translated questionnaire to ensure these two versions were identical in meaning. The final translated items were piloted on 10 undergraduate students with all participants reporting no misunderstanding.

#### Acceptance and Action Questionnaire II

The seven-item Acceptance and Action Questionnaire II (AAQ-II) was used to measure EA (Bond et al., 2011). The response scale has a range of 1 (never true) to 7 (always true), a higher score indicated a higher degree of EA. From the initial validation study (α = 0.88) and another in college students in mainland China (α = 0.84), a good internal consistency was observed (Cao et al., 2013). In the current sample, AAQ-II also demonstrated good internal consistency (Cronbach’s α = 0.87).

#### Mindful Attention Awareness Scale

Present-moment awareness can be considered a fundamental component of mindfulness and it is most commonly measured with the 15-item Mindful Attention Awareness Scale (MAAS) (Brown and Ryan, 2003). It uses a seven-point scale from 1 (almost always) to 6 (almost never). Good internal reliability (α = 0.89; MacKillop and Anderson, 2007) was shown in the original validation study, and in a Chinese student sample (α = 0.85; Deng et al., 2011). In the current sample, MAAS also demonstrated good internal consistency (α = 0.87).

#### Depression Anxiety and Stress Scale-21

Mental distress was measured by Depression Anxiety and Stress Scale-21 (DASS-21), which includes 21 items with three subscales reflecting three types of negative states: anxiety, depression, and stress (Lovibond and Lovibond, 1995). A four-point Likert scale was used to assess items, running from 0 (did not apply to me at all) to 3 (applied to me very much, or most of the time). Good internal reliabilities for the anxiety, depression, and stress subscales (α = 0.87–0.94) were shown in the original version (Antony et al., 1998) and the Chinese version (α = 0.76–0.79) (Gong et al., 2010). The reliability coefficients were 0.83 (depression), 0.78 (anxiety), and 0.81 (stress) in the current study.

#### Multidimensional Students’ Life Satisfaction Scale

Life satisfaction of students was evaluated with the 40-item Multidimensional Students’ Life Satisfaction Scale (MSLSS) across five domains of family, friends, school, surroundings, and self (Huebner et al., 1998). Questions were rated using a single six-point Likert-type scale from 1 (disagree completely) to 6 (agree completely). Overall MSLSS score and five domain scores were calculated. Good internal reliability for the total scale (α = 0.91) was shown in the original validation study, and in a Chinese student sample (α = 0.90) (Tian and Liu, 2005). The reliability coefficient for the total scale was 0.92 in this study.

### Statistical Analysis

Statistical analysis consisted of three parts. First, preliminary data analyses using descriptive statistics were conducted. Second, to ensure that the psychometric properties of the Mandarin Chinese version of the CAQ-8 were adequate, reliability, and validity analyses were conducted using IBM SPSS Statistics 24.0. Homogeneity was evaluated using corrected item-total correlations. To determine reliability, Cronbach’s alpha was used for internal consistency, and test–retest reliability used intraclass correlation coefficients (ICCs) based on a model of two-way random effects (Koo and Li, 2016).

Based on theory and previous research on the CAQ-8 (McCracken, 2013; Trompetter et al., 2013; McCracken et al., 2015; Gagnon et al., 2016; Wong et al., 2016; Trindade et al., 2018), two competing models were derived: a correlated two-factor model (two-factor model) and a general factor plus two subsidiary factors model (bifactor model). In the correlated two-factor model, every item was assigned to load on one of two factors, anticipated to represent the two subscales, with the two factors allowed to be correlated. The general factor plus two subsidiary factors model (bifactor model) hypothesized that CAQ-8 could be explained by three potential factors, namely, a general factor standing for the committed action and two factors representing the subscales. These three factors would not correlate with each other. We conducted confirmatory factor analysis (CFA) to examine which model best fitted the Chinese population. Maximum likelihood estimation was employed for CFA, using Mplus 8.0, to examine the factor structure. Since chi-square is sensitive to sample size, four commonly used indices were observed to test model fit. The Tucker–Lewis index (TLI) and comparative fit index (CFI) should be above a recommended level of 0.90 to indicate good fit, while the standardized root-mean-square residual (SRMR) and the root-mean-square error of approximation (RMSEA) are acceptable when inferior to 0.08 (Hu and Bentler, 1999).

Convergent validity was investigated through the relationship between the CAQ-8 (Mandarin) and remaining theoretically linked measures, such as EA (AAQ-II) and present-moment awareness (MAAS), which are measures of psychological flexibility that originated from ACT and should conceptually be related. In addition, previous studies have found committed action to be a predictor of both mental distress (Gagnon et al., 2017; Trindade et al., 2018) and life satisfaction (Coutinho et al., 2019). Therefore, DASS-21 and MSLSS were used as measures of the criterion validity, expecting that CAQ-8 would be negatively correlated with mental distress, and positively correlated with life satisfaction. Additionally, hierarchical regression analyses were performed to assess the incremental validity of the CAQ-8 above that of the AAQ-II and MAAS as measures of related ACT processes, with depression, anxiety, stress, and MSLSS as dependent variables. In the first step, the AAQ-II and MAAS total scores were entered to predict the dependent variables. In the second step, the CAQ-8 total score was added. The incremental validity was evaluated by the change in variance from the second step.

The third part of the statistical analysis is the test of mediating effect. SPSS PROCESS was used to perform the path analyses (Hayes, 2013) to verify mediating effects. We tested Model 4, Hayes’ SPSS PROCESS model (Hayes, 2013), in which EA (AAQ-II) is the predictor, life satisfaction (MSLSS) the dependent variable, and committed action (CAQ) the mediator. We used bootstrapping with 5,000 samples to calculate the 95% CIs and the bias-corrected method of the indirect effects in all statistical analyses (Hayes, 2013). Based on the bootstrapping methods, the mediating effect was considered significant if the 95% the bias corrected confidence interval (BCCI) did not overlap with 0 (Hayes, 2013).

## Results

### Descriptive Statistics

The demographic characteristics of the participants are shown in Table 1. CAQ-8 in the “Results” section refers to the Mandarin version. The final sample with no missing data consisted of 913 participants, 268 men (29.4%) and 645 women (70.6%). No sex difference was found for the total scores of CAQ-8 (*t* = –1.09, *p* = 0.28).

**TABLE 1:** Demographic characteristics of the participants (*n* = 913).

Variable	Classification	*n*	%	*M* (SD)	Range
Age		913		18.84 (1.25)	16–25
Sex	Male	268	29.4%		
	Female	645	70.6%		
Ethnicity	Han	795	87.1%		
	Non-Han	118	12.9%		
Residence	Countryside	611	66.9%		
	City	288	31.5%		
	Others	14	1.5%		

For CAQ-8, the mean scores were 29.90 ± 7.04 (total scale), 15.10 ± 4.39 (VP subscale), and 9.20 ± 4.62 (NB subscale). The skewness of CAQ-8 items varied between –0.66 and –0.05 and kurtosis varied between –0.58 and 0.10, all within the recommended range with absolute values not above 3 and 10, respectively (Kline, 2005). These findings suggest that the distributions of all items were relatively normal. Four weeks later, 167 participants fully completed the CAQ-8 a second time to evaluate test–retest reliability, the mean score was 31.01 ± 5.71.

### Reliability

The homogeneity of the CAQ-8 was evaluated by examining corrected item-total correlations, and the results indicated that all of the item-total correlations were greater than the threshold of 0.30 (Nunnally and Bernstein, 1994) and had a range from 0.48 to 0.67. The Cronbach’s alpha coefficients for the overall scale, NB subscale, and VP subscale were 0.77, 0.75, and 0.84, respectively, suggesting moderately high internal consistency, which surpassed acceptable α levels of between 0.60 and 0.70 (DeVellis, 1991). The ICC was used according to Shrout and Fleiss (1979) as an estimation of the test–retest reliability index. By Shrout’s (Shrout, 1998) criteria, below 0.40 was considered poor, 0.41–0.60 ordinary, 0.61–0.80 moderate, and above 0.80 good. The test–retest reliability coefficients (*n* = 167) had ICC values of 0.79 for CAQ-8 total, 0.68 for VP subscales, and 0.68 for the NB subscales. These findings suggest moderate test–retest reliability for the CAQ-8.

### Validity

#### Structure Validity

Two competing models, a two-factor model and a bifactor model, were tested. Table 2 shows that only the two-factor model achieved an acceptable fit based on CFI, TLI, RMSEA, and SRMR for the present sample. The structure validity was supported by the two-factor model, which indicated that the items of CAQ-8 comprise two correlated factors. Table 3 reports the results of factor loadings, which exceeded the 0.4 cut-off value ranging from acceptable to good (Tabachnick and Fidell, 2007). The results were in line with the original scale.

**TABLE 2:** Confirmatory factor analysis for two competing measurement models.

Model	S-B χ^2^	*df*	CFI	TLI	RMSEA	SRMR
Bifactor model	72.759	15	0.968	0.939	0.065	0.126
Two-factor model	53.581	19	0.981	0.972	0.045	0.036

**TABLE 3:** Factor loadings of the Committed Action Questionnaire (CAQ-8) items.

Latent factor	Items	Standardized factor loading coefficients
Factor 1 Values persistence	I can remain committed to my goals even when there are times that I fail to reach them	0.813
	When a goal is difficult to reach, I am able to take small steps to reach it	0.761
	I prefer to change how I approach a goal rather than quit	0.755
	I am able to follow my long term plans including times when progress is slow	0.678
Factor 2 Non-reactive behavior	I find it difficult to carry on with an activity unless I experience that it is successful	0.640
	If I feel distressed or discouraged, I let my commitments slide	0.746
	I get so wrapped up in what I am thinking or feeling that I cannot do the things that matter to me	0.690
	If I cannot do something my way, I will not do it at all	0.535

#### Convergent and Criterion Validity

Convergent validity of CAQ-8 scale scores was assessed by Pearson correlations between committed action and theoretically correlated constructs such as EA (AAQ-II) and present-moment awareness (MAAS). As shown in Table 4, the total score of CAQ-8 was moderately correlated with the AAQ-II (*r* = –0.43, *p* < 0.001) and the MAAS (*r* = 0.43, *p* < 0.001), which measured EA and present-moment awareness, respectively.

**TABLE 4:** Correlations between CAQ-8 and relevant variables.

	1	2	3	4	5	6	7	8	9
(1) CAQ–8	1								
(2) VP	0.77***	1							
(3) NB	–0.79***	–0.22***	1						
(4) MAAS	0.43***	0.26***	-0.40***	1					
(5) AAQ-II	–0.43***	–0.24***	0.43***	–0.62***	1				
(6) Depression	–0.38***	–0.25***	0.35***	–0.54***	0.46***	1			
(7) Anxiety	–0.32***	–0.21***	0.29***	–0.58***	0.51***	0.77***	1		
(8) Stress	–0.35***	–0.20***	0.34***	–0.62***	0.55***	0.75***	0.82***	1	
(9) MSLSS	0.42***	0.39***	–0.27***	0.35***	–0.36***	–0.53***	–0.42***	–0.43***	1

***p < 0.001 (two-tailed).

*CAQ-8, the Committed Action Questionnaire; VP, values persistence; NB, non-reactive behavior; MAAS, Mindful Attention Awareness Scale; AAQ-II, Acceptance and Action Questionnaire; MSLSS, Multidimensional Students’ Life Satisfaction Scale.*

Criterion validity was evaluated by calculating the association between the CAQ-8 and measures of mental distress (DASS-21) and life satisfaction (MSLSS). The results showed that the total score of CAQ-8 was significantly negatively correlated with depressive symptoms (*r* = –0.38, *p* < 0.001), anxiety (*r* = –0.32, *p* < 0.001), stress (*r* = –0.35, *p* < 0.001), and positively correlated with life satisfaction (*r* = 0.42, *p* < 0.001), as expected, supporting the criterion validity of the CAQ-8.

Table 5 shows the hierarchical regression analyses. The CAQ-8, when added to the models, added significant variance to the prediction of depression and life satisfaction, but not for the prediction of anxiety and stress.

**TABLE 5:** Hierarchical multiple regression predicting depression, anxiety, stress, and life satisfaction.

Block		Depression				Anxiety				Stress				Life satisfaction		
		β	*t*	adj *R*^2^		β	*t*	adj *R*^2^		β	*t*	adj *R*^2^		β	*t*	adj *R*^2^
1	AAQ-II	0.20	5.70**	0.312**	1	0.25	7.40**	0.372***	1	0.26	8.18**	0.421**	1	–0.23	–5.96**	0.150**
	MAAS	–0.41	–11.79**			–0.43	–12.70**			–0.45	–14.05**			0.20	5.14**	
2	AAQ-II	0.16	4.42**	0.330**	2	0.24	6.86**	0.373	2	0.25	7.54**	0.422	2	–0.15	–3.89**	0.220**
	MAAS	–0.37	–10.54**			–0.42	–12.08**			–0.44	–13.33**			0.12	3.22**	
	CAQ-8	–0.15	–4.99**			–0.04	–1.37			–0.05	–1.74			0.30	9.05**	

**p < 0.01 (two-tailed).

*CAQ-8, the Committed Action Questionnaire; MAAS, Mindful Attention Awareness Scale; AAQ-II, Acceptance and Action Questionnaire; MSLSS, Multidimensional Students’ Life Satisfaction Scale.*

### Mediation Analysis

As shown in Table 4, EA was correlated with committed action, and both committed action and EA were correlated with life satisfaction. This aligns with previous findings (Kashdan et al., 2006; Chawla and Ostafin, 2007; Graham et al., 2016; Valdivia-Salas et al., 2017; Trindade et al., 2018; Lucas and Moore, 2020). On this basis, we further investigated whether committed action mediates the relationships between EA and life satisfaction. In the hypothesized mediation model, EA was the independent variable, committed action was the mediating variable, and life satisfaction was the dependent variable. A mediator model was used to test if committed action mediates the association between EA and life satisfaction.

**FIGURE 1:**
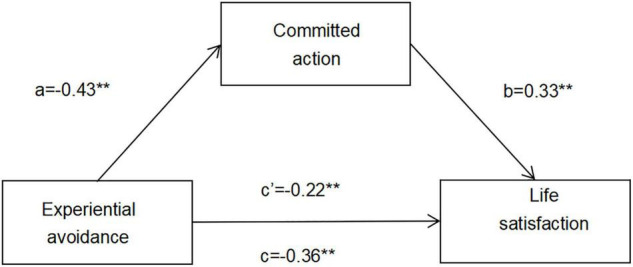
The hypothesized mediation model in which experiential avoidance is associated with life satisfaction directly and indirectly *via* committed action. Standardized coefficients (β) are presented. **p < 0.01.

As shown in Figure 1, (a) EA had a significant negative effect on committed action (β = –0.43, SE = 0.03, and *p* < 0.001); (b) committed action had a positive effect on life satisfaction (β = 0.33, SE = 0.02, and *p* < 0.001); (c) EA had a direct and significant negative effect on life satisfaction (β = –0.36, SE = 0.01, and *p* < 0.001), which indicated that participants scoring higher on EA tended to score lower on committed action and life satisfaction; and (d) after the mediating variable of committed action was controlled, the influence of EA on life satisfaction was still significant, but was lower than the unmediated effect (β = –0.22, SE = 0.01, and *p* < 0.001). As shown in Table 6, a significant mediating effect of committed action was discovered with an indirect effect of –0.060 [SE = 0.008, 95% BCCI (–0.077, –0.046); *p* < 0.001], which accounted for 39.22% of the total influence of EA on life satisfaction.

**TABLE 6:** Results of the mediated path analysis.

	Effect	Boot SE	Bootstrapping		Ratio of indirect to total effect
			BC 95% CI		
			Lower	Upper	
Total effect	–0.153	0.015	–0.182	–0.123	
Direct effect	–0.093	0.031	–0.122	–0.065	
Indirect effect	–0.060	0.008	–0.077	–0.046	39.22%

*Total effect, effect of experiential avoidance on life satisfaction; indirect effect, effect of experiential avoidance on life satisfaction through committed action; direct effect, effect of experiential avoidance on life satisfaction after controlling for committed action; boot SE, were estimated standard error; BC, bias-corrected; 95% CIs do not include.*

## Discussion

Committed action refers to goal-directed behaviors with flexible persistence and is a core process of psychological flexibility. The development of a Mandarin Chinese version of the CAQ-8 may contribute to the development of an in-depth understanding of committed action in mainland China. This study provides preliminary evidence for the reliability and validity of the Mandarin Chinese version of the CAQ-8. Alpha levels for the CAQ-8 and its subscales ranged from 0.75 to 0.84 and were similar to those of the English CAQ (Gagnon et al., 2017) and ChCAQ-8 (Galán et al., 2019). The test–retest reliability, measured by ICC also found acceptable stability. With regards to the factor structure, CFA supported a two-factor model, as with the original questionnaire, providing initial support for its use in China and cross-culturally more generally.

Regarding convergent validity, the CAQ-8 score was significantly correlated with EA assessed by AAQ-II and present-moment awareness assessed by MAAS, which are theoretically related constructs. In line with expectations, higher levels of committed action were associated with lower levels of EA and higher mindful awareness. Criterion validity was confirmed by correlations between committed action and depression, stress, anxiety, and life satisfaction. The results suggested that higher levels of committed action were correlated with lower levels of mental distress and greater life satisfaction. The results of the hierarchical regression analyses suggest that the CAQ-8 score added incremental variance in addition to AAQ-II and MAAS when predicting depression and life satisfaction, but did not do so for anxiety and stress. One previous study found that low committed action is a predictor of depression (McCracken, 2013). A previous study found that psychological flexibility was a strong predictor of life satisfaction (Lucas and Moore, 2020), suggesting that living in accordance with personal values increased life satisfaction. Another recent study found that committed action, as one core component of psychological flexibility, could mitigate the adverse effects of trait health anxiety on mental health during COVID-19 lockdown in Italy (Landi et al., 2020). There does not appear to be any previous research exploring the effect of committed action on stress. Here, CAQ-8 scores did not contribute significant variance in predicting anxiety or stress. This may be because committed action can occur even in the presence of distress (McCracken, 2013), such as worry (anxiety) or stress. Besides, CAQ-8, MAAS, and AAQ-II, as measures of related ACT processes, may share a common variance for explaining stress and anxiety.

Overall, these findings suggest that the Mandarin Chinese version of the CAQ-8 is adequate for use in a Chinese university student sample. So, the current study expands the use of the CAQ-8 in a non-clinical population and lays the foundation for future cross-cultural studies.

In addition, these findings extend previous research by exploring the mediating roles of committed action in the association between EA and life satisfaction. The direct impact of EA on life satisfaction is well-established (Palladino et al., 2013; Graham et al., 2016; Valdivia-Salas et al., 2017; Trindade et al., 2018; Lucas and Moore, 2020), although the mechanisms are relatively unclear. Here, it appears that committed action partially mediated the effect of EA on life satisfaction. This suggests that being more prone to EA—to be unwilling to accept unwanted inner experience—is related to the reduced committed action, which, in turn, reduces life satisfaction (Trompetter et al., 2013). This suggests that being open to unpleasant private experiences and engaging in activities based on personal values and goals are significantly related to greater life satisfaction. Theoretically, the ability to pursue valued goals in the presence of unwanted private events has a positive impact on life satisfaction (Graham et al., 2016; Graham and Rose, 2017).

Findings also provide more general support for psychological flexibility theory, which emphasizes the need to pursue valued goals in the context of acceptance to live an ideal life, which would be one way of defining life satisfaction (Diener, 1984). People engaged in EA may disrupt committed action so, despite avoidance reducing upset in the short term, it increases suffering and generates behavior inflexibility in the long run, thereby preventing the person from engaging in wellbeing enhancing activities (Hayes et al., 1996). In contrast, committed action includes flexible persistence so that people can adapt actions when goal-directed behavior leads to pain, distress, and failure (McCracken, 2013).

There are some limitations of the study. First, the sample was comprised of university students. Further studies should be expanded to the general Chinese population and clinical samples. Second, convenience sampling produced a predominantly female sample. Third, the mediation analysis was cross-sectional. A longitudinal design test for mediation effects would be more rigorous and powerful (Cole and Maxwell, 2003). Fourthly, the measure of life satisfaction used was designed for children and adolescents, which may limit the translation of these results to older groups.

## Conclusion

The CAQ-8 (Mandarin) appears to be a promising measurement tool for assessing committed action among a sample of Chinese students, which could also be used in the general population. More generally, the psychological flexibility model and ACT appear to apply in China. Moreover, committed action may partially mediate the association between EA and life satisfaction, suggesting that committed action could play a significant role in explaining how EA is related to life satisfaction.

## Data Availability Statement

The raw data supporting the conclusions of this article will be made available by the authors, without undue reservation.

## Ethics Statement

The studies involving human participants were reviewed and approved by The Third Xiangya Hospital of Central South University (2020-S385). The patients/participants provided their written informed consent to participate in this study.

## Author Contributions

YL and Q-PT were involved in methodology and wrote the manuscript with contributions from all the co-authors. F-LY and CP obtained funding and contributed to formal analysis and project administration. Q-QC was involved in the investigation. All authors have participated in the conceptualization of the research, contributed to the article, and approved the submitted version.

## Conflict of Interest

The authors declare that the research was conducted in the absence of any commercial or financial relationships that could be construed as a potential conflict of interest.

## Publisher’s Note

All claims expressed in this article are solely those of the authors and do not necessarily represent those of their affiliated organizations, or those of the publisher, the editors and the reviewers. Any product that may be evaluated in this article, or claim that may be made by its manufacturer, is not guaranteed or endorsed by the publisher.

## Abbreviations

CAQ-8: the Committed Action Questionnaire
VP: values persistence: positive subscale of the Committed Action Questionnaire
NB: non-reactive behavior: negative subscale of the Committed Action Questionnaire
MAAS: Mindful Attention Awareness Scale
AAQ-II: Acceptance and Action Questionnaire
MSLSS: Multidimensional Students’ Life Satisfaction Scale
EA: experiential avoidance.
